# Sc-Decorated Porous Graphene for High-Capacity Hydrogen Storage: First-Principles Calculations

**DOI:** 10.3390/ma10080894

**Published:** 2017-08-02

**Authors:** Yuhong Chen, Jing Wang, Lihua Yuan, Meiling Zhang, Cairong Zhang

**Affiliations:** 1 State Key Laboratory of Advanced Processing and Recycling of Non-Ferrous Metals, Lanzhou University of Technology, Lanzhou 730050, China; mirror1217@163.com (J.W.); zhcrxy@lut.cn (C.Z.); 2 School of Science, Lanzhou University of Technology, Lanzhou 730050, China; yuanlh@lut.cn (L.Y.); zhangml_2000@126.com (M.Z.)

**Keywords:** first principles, Sc modification, porous graphene, hydrogen storage

## Abstract

The generalized gradient approximation (GGA) function based on density functional theory is adopted to investigate the optimized geometrical structure, electron structure and hydrogen storage performance of Sc modified porous graphene (PG). It is found that the carbon ring center is the most stable adsorbed position for a single Sc atom on PG, and the maximum number of adsorbed H_2_ molecules is four with the average adsorption energy of −0.429 eV/H_2_. By adding a second Sc atom on the other side of the system, the hydrogen storage capacity of the system can be improved effectively. Two Sc atoms located on opposite sides of the PG carbon ring center hole is the most suitable hydrogen storage structure, and the hydrogen storage capacity reach a maximum 9.09 wt % at the average adsorption energy of −0.296 eV/H_2_. The adsorption of H_2_ molecules in the PG system is mainly attributed to orbital hybridization among H, Sc, and C atoms, and Coulomb attraction between negatively charged H_2_ molecules and positively charged Sc atoms.

## 1. Introduction

Considerable research has been conducted to search for clean, renewable energy to meete growing energy demand [1]. Hydrogen has many advantages such as being recyclable, highly abundant, pollution-free, of high gravimetric energy density, and high combustion heat. Therefore hydrogen is considered to be an ideal carrier for energy storage [2]. However, the primary obstacle to hydrogen application is safe and efficient storage under ambient conditions [3,4]. To meet the criteria of the U.S. Department of Energy (DOE) and the International Energy Agency (IEA), the desirable hydrogen storage capacity of a promising material should be greater than 5.5 wt %. The adsorption energy between H_2_ molecules and materials should be from 0.2 to 0.7 eV [5]. Therefore, it is appropriate that hydrogen stores in solid materials in a molecular form [6].

Carbon nanostructures, such as graphene [7,8], fullerene [9,10], and nanotubes [11] have been investigated for solid-state hydrogen storage because of their high specific surface area, fast kinetics, and reversible hydrogen storage etc. However, H_2_ molecules bind weakly with pristine graphene, which is chemically too inert to act as a promising hydrogen storage material. Many studies have been performed to improve the chemical activity of graphene, such as doping or decoration by alkali metals [12,13], alkaline-earth metals [14,15,16], and transition metals [17,18,19]. The adsorption energy of hydrogen molecules on graphene can significantly be improved by the modification of transition metal (TM) atoms [20]. However, the large cohesive energy of TM atoms can easily lead to the formation of clusters on graphene. Research shows that the introduction of impurity atoms or vacancies can prevent the TM atoms from aggregating, thus increasing the hydrogen storage capacity of graphene [21]. Therefore, graphene which has a foraminiferous defect and doped atoms, decorated with TM atoms, has attracted attention.

Luo et al. [22] noted that the Sc-decorated single-vacancy defect graphene with three N atoms doped was the best medium for hydrogen storage, using Vienna Ab-initio Simulation Package (VASP) code within the GGA functional calculation, because each Sc atom could adsorb five H_2_ molecules with the adsorption energies from 0.2 to 0.4 eV. Using DMol^3^ code within the local density approximation (LDA) functional calculation, Omar Faye et al. [23] found that up to eight H_2_ molecules could be adsorbed by double-sided Pd-functionalized graphene. In addition, Omar Faye et al. [24] also studied hydrogen storage in Cu-functionalized boron-doped graphene by the same method. A gravimetric hydrogen density of 4.231 wt % was reached when H_2_ adsorbed on double-sided Cu-functionalized graphene. Using the DMol^3^ package within the LDA functional calculation, Y decorated B-doped graphene was studied as a potential carrier for hydrogen storage, in which the hydrogen storage capacity was 5.78 wt %. The corresponding average adsorption energy was −0.568 eV per H_2_ molecule [25]. Although the average adsorption energy of H_2_ molecules is suitable for reversible hydrogen storage in these systems, the hydrogen storage capacity is close to the minimum 5.5 wt % of United States DOE.

Because of the good performance of graphene, other graphene-like materials have also attracted attention. Examples are silicene [26,27,28], monolayer black phosphorus [29], arsenene [30] and porous graphene [31]. Porous graphene (PG) was successfully fabricated by Bieri et al [32]. The inherent nanopores in porous graphene are well-developed and uniformly distributed, which portend highly effective hydrogen storage by a high specific surface area [33]. Du et al. [12] investigated Li-decorated PG by VASP code within the LDA, which found that the hydrogen storage capacity was up to 12 wt %. However, the average binding energy of 0.243 eV per H_2_ was overestimated due to the LDA function. Reunchan et al. [34] investigated the Ca-decorated PG and found that an isolated Ca atom could adsorb five hydrogen molecules with the average adsorption energy of 0.230 eV. Recently, Yuan et al. [35] studied Y-decorated porous graphene and they found that the maximum number of adsorbed hydrogen molecules around the Y atom was six and the average adsorption energy was −0.297 eV.

Sc is the lightest TM atom, indicating that Sc-decorated PG could produce higher hydrogen storage capacity. In this paper, we calculate the most stable adsorption structure of Sc atoms on PG and the corresponding adsorption energy. In addition, the adsorption properties and adsorption mechanism of H_2_ molecules on the Sc decorated PG system are analyzed to better understand the effect of Sc atoms modified PG on the hydrogen storage properties.

## 2. Calculation Details

Our studies are performed using the plane wave CASTEP package [36] with the Perdew-Burke-Ernzerhof (PBE) of the generalized gradient approximation (GGA) exchange-correlation function [37]. Since the GGA function may underestimate the relatively weak adsorption energies, a pragmatic method to resolve this issue has been given by the dispersion-corrected density functional theory (DFT-D) approach. Calculations are performed using the DFT-D method to consider the van der Waals forces. All the atoms are relaxed in our calculations such that the force on each atom is less than 0.01 eV/Å. The convergence tolerance energy is 5.0 ×$\;10^{-6}$ eV/atom to realize the energy minimization. The self-consistent field (SCF) convergence threshold is 1.0 ×$\;10^{-6}$ eV/atom. The calculation of the porous graphene unit cell satisfies the periodic boundary conditions. The vacuum thickness of 20 Å is adopted to minimize the interlayer interaction. In order to ensure convergence and improve the accuracy of our calculations, the cutoff energy and the k-point are tested. Considering the test results and the computational cost, the cutoff energy is set to 500 eV and the k-point is set to 7 × 7 × 1.

The average adsorption energy of Sc atoms on PG layer is defined as:

(1) $${\bar{E}}_{b}=\left[E_{\mathrm{Sc}+\mathrm{PG}}-E_{\mathrm{PG}}-nE_{\mathrm{Sc}}\right]/n$$

where $E_{\mathrm{Sc}+\mathrm{PG}}$, $E_{\mathrm{PG}}$ and $E_{\mathrm{Sc}}$ represent the total energy of the PG system with Sc atoms, the total energy of PG and the total energy of a free Sc atom, respectively. Moreover, *n* represents the number of adsorbed Sc atoms.

The adsorption energy $\left(E_{\mathrm{ad}}\right)$ and average adsorption energy (${\bar{E}}_{\mathrm{ad}}$) of adsorbed H_2_ molecules are respectively defined as:

(2) $$E_{\mathrm{ad}}=\left[E_{iH_{2}+\mathrm{Sc}+\mathrm{PG}}-E_{(i-1)H_{2}+\mathrm{Sc}+\mathrm{PG}}-E_{H_{2}}\right]$$

(3) $${\bar{E}}_{\mathrm{ad}}=\left[E_{iH_{2}+\mathrm{Sc}+\mathrm{PG}}-E_{\mathrm{Sc}+\mathrm{PG}}-iE_{H_{2}}\right]/i$$

where $E_{iH_{2}+\mathrm{Sc}+\mathrm{PG}}$ and $E_{(i-1)H_{2}+\mathrm{Sc}+\mathrm{PG}}$ are the total energy of the Sc-decorated PG system with i and (i−1) H_2_ molecules adsorbed, respectively. $E_{H_{2}}$ represents the energy of an isolated H_2_ molecule.

**Figure 1:**
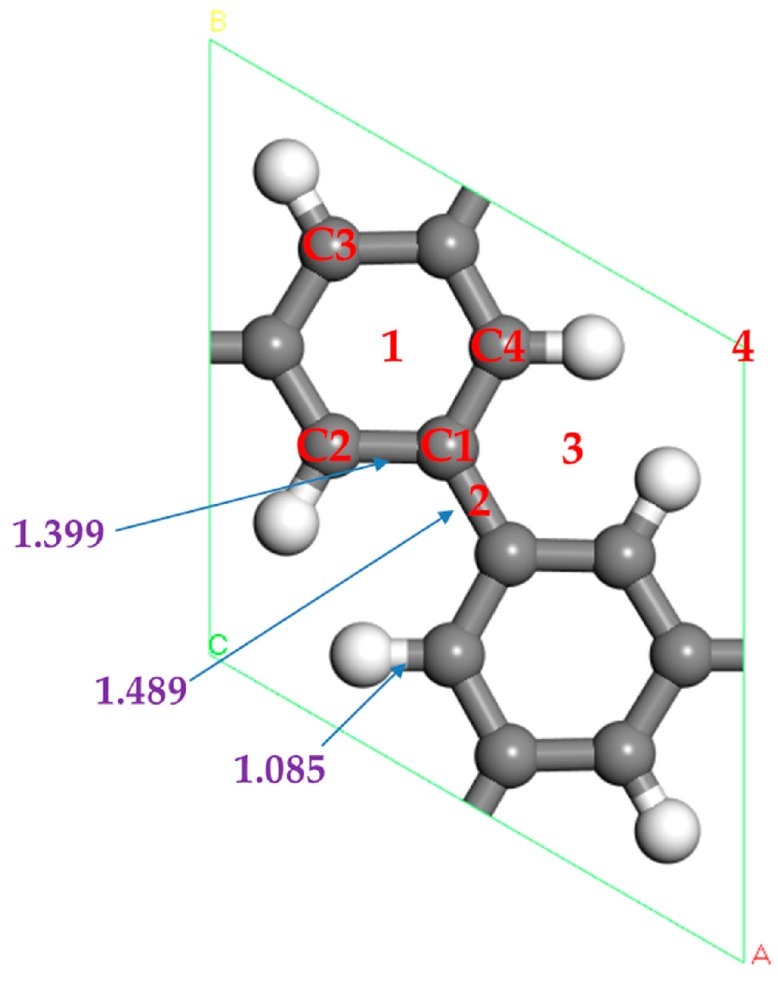
The optimized atomic structure of porous graphene. The gray and white balls represent C and H respectively.

The fully optimized structure of a porous graphene unit cell is shown in Figure 1. The calculated lattice constant is 7.49 Å, which agrees well with the experimental value of 7.40 Å [32]. There are two kinds of C atoms on PG. C1 is connected with three C atoms, while C2 is connected with two C atoms and one H atom. The bond length of C1–C1 is 1.489 Å, C1–C2 is 1.399 Å, C2–H is 1.085 Å. Our results are very close to the values of Brunetto [38], who gets the results using the DMol^3^ package with the GGA-BLYP function. In addition, Li [39] calculates the bond length of C2–H at 1.080 Å using the DMol^3^ package with the GGA-PW91 function. The result is consistent with our value of 1.085 Å. The direct band gap is calculated to be 2.399 eV using the GGA with the PBE function, which coincides well with the results of Rao [37] who obtains the value of band gap at 2.400 eV based on VASP code with the PBE of the GGA function. The above test results show that the calculation method and parameters used in this paper are reliable.

## 3. Results and Discussion

### 3.1. Single Sc Atom Decorated PG

#### 3.1.1. The Adsorption Structure of Single Sc Atom Decorated PG

**Figure 2:**
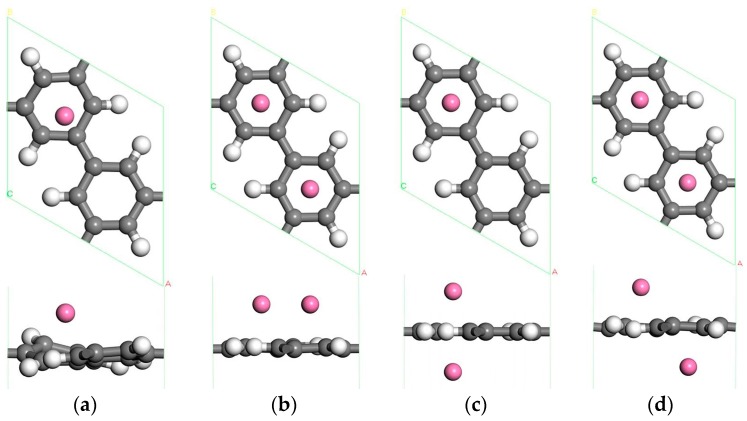
The optimized atomic structure of Sc atom decorated PG. (**a**) single Sc atom decorated PG; (**b**) two Sc atoms decorated single-sided PG; (**c**) two Sc atoms decorated double-sided PG at the same hole; (**d**) two Sc atoms decorated double-sided PG at the adjacent hole. The gray, white and pink balls in this and following figures denote C, H and Sc atoms, respectively.

Now we study the adsorption of one Sc atom on PG. There are four different symmetric positions for a Sc atom adsorption, which are shown in Figure 1 and named 1, 2, 3, 4, indicating the hollow center of C ring, the bridge of C–C bond, the hollow center of a half C ring, and a large hexagonal pore, respectively. It is found that the 1 position is the favorite adsorption site for a Sc atom on PG, and this position is the same as the adsorption of a Sc atom on graphene [22]. The favorite configuration after full relaxation is shown in Figure 2a. We find that the Sc atom is a little deviation from the hollow center of the C-hexagon, and the adsorption energy is −2.143 eV for a Sc atom on PG.

**Figure 3:**
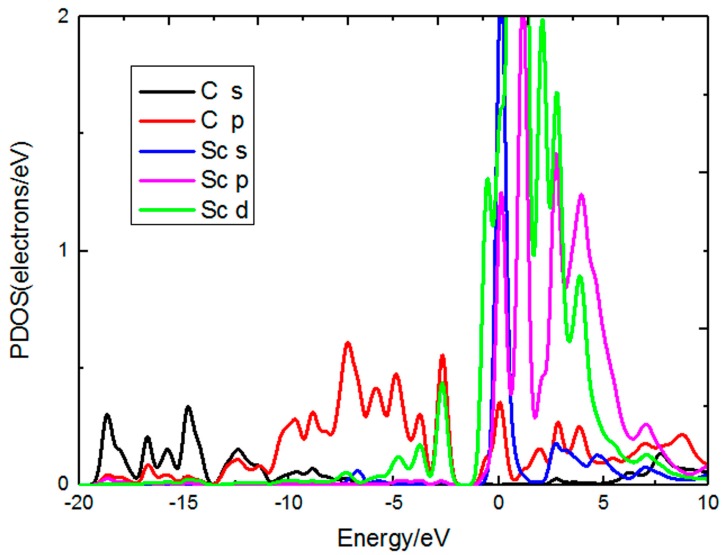
PDOS of Sc-decorated PG system.

We also calculate the adsorption of Sc on perfect graphene to further investigate the effect of graphene defects on Sc adsorption. The adsorption energy of the Sc atom on the intact graphene is −1.499 eV, which is calculated using the CASTEP package within the PBE of the GGA function. This is very close to the value of −1.550 eV, which is calculated based on VASP code with the PBE of the GGA function [22]. The adsorption energy of Sc atoms on perfect graphene is far less than that on PG. After adsorption, the positive charges of the Sc atom on the perfect graphene and PG are 1.24 e, 1.33 e, respectively. In other words, more charge transfers in the Sc-decorated PG system, and this stems from the fact that the interaction between Sc atoms and PG is stronger than the interaction between Sc atoms and perfect graphene. From the partial densities of states (PDOS) of Sc and C in Figure 3, we can learn the peak of the C 2p orbital overlap with the peak of the Sc 3d orbital at −2.50 eV, and this suggests a strong hybridization between C and Sc atoms.

#### 3.1.2. The Adsorption of H_2_ Molecules on Single Sc-Decorated PG

**Figure 4:**
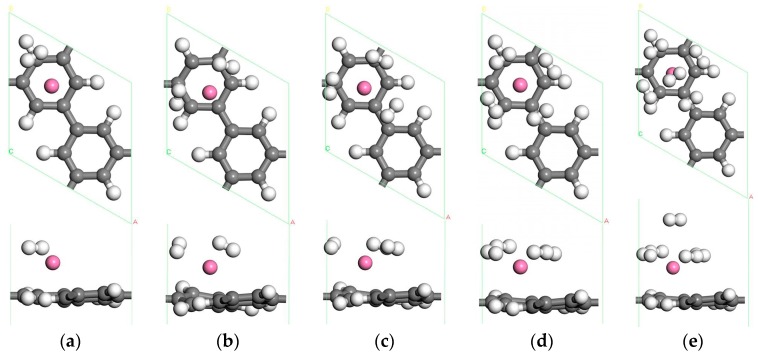
The optimized atomic structures of the Sc atom decorated PG with (**a**) one H_2_ molecule; (**b**) two H_2_ molecules; (**c**) three H_2_ molecules; (**d**) four H_2_ molecules; (**e**) five H_2_ molecules adsorbed.

The optimized geometries for H_2_ molecules adsorbed on the Sc-decorated porous graphene are shown in Figure 4, and Table 1 lists the adsorption energy and the average adsorption energy of H_2_ molecules. There are several positions for single H_2_ molecule adsorption, including the bridge of the C–C bond, the bridge of the C–H bond, the top of the C atom and the top of the Sc atom. It is found that the favorite position for the first H_2_ molecule is above the C–H bond. The binding energy is −0.401 eV, and the bond length is elongated to 0.818 Å. In order to study the hydrogen storage capacity of the system, we add other H_2_ molecules to the system one by one. From Table 1, we can see that the adsorption energy of H_2_ molecules first increases and then decreases for the consecutive adsorption of the H_2_ molecule. However, $d_{\mathrm{Sc}-C}\;$(the nearest distance between the Sc atom and the C atom of porous graphene) is the opposite, which means that the adsorption of H_2_ molecules influences the binding between the Sc atom and porous graphene. It is very interesting to note that almost all H_2_ molecules are symmetrically distributed around the Sc atom because of the symmetry of the bonding configuration of H_2_ molecules. In addition, the first four H_2_ molecules are parallel to the PG sheet and stay on the same plane. When the fifth H_2_ molecule is added to the system, it moves to an upper layer after relaxation, as shown in Figure 4e. This effect may be due to the limited space around the Sc atom and the repulsive interaction between the adsorbed H_2_ molecules [40]. Therefore, the H–H bond length of the fifth H_2_ molecule is 0.756 Å and this H_2_ molecule has the smallest adsorption energy of −0.093 eV, which is weak for hydrogen storage application [41]. The system of the single Sc atom decorated porous graphene can adsorb a maximum of 4 H_2_ molecules because of the two-layer arrangement. Therefore, the hydrogen storage capacity is 3.94 wt % and the average adsorption energy is −0.429 eV/H_2_, which is higher than that of Sc modified defect graphene with three N atoms doped [22].

**Figure 5:**
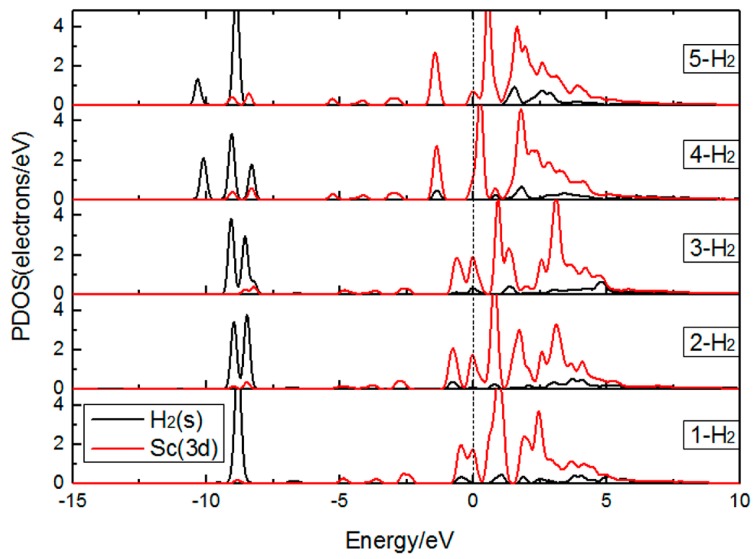
PDOS of Sc-decorated PG with one to five H_2_ molecules adsorbed.

In order to investigate the interaction between the Sc atom and adsorbed H_2_ molecules, the partial densities of states for the H_2_ molecules and the Sc atom are plotted in Figure 5. It can be observed that there is a band broadening around −9 eV, which indicates an H_2_–H_2_ interaction. In addition, there is a strong hybridization between H_2_ 1s orbitals and Sc 3d orbitals around −10.5 to −8.0 eV, since the orbital overlap is between 1s of H_2_ and 3d of Sc. In the range −1.5 to 0 eV, the adsorption of 1~4 H_2_ molecules can be seen to have overlapping peaks between H_2_ 1s orbital and Sc 3d orbital, suggesting that in this interval H_2_ molecules and the Sc atom also exist in hybridization, which may be one of the reasons why the first four H_2_ molecules have a strong interaction with the Sc atom. From the PDOS of H_2_ molecules, we can see that the 1s orbital mainly distributes in σ bonding state, namely a bonding state dominates the 1s orbital and there is no H_2_ molecular dissociation.

Given the Mulliken charge population before and after H_2_ molecules adsorption, the bond strength and charge transfer between atoms could be analyzed. The Mulliken population analysis of Sc-PG system with and without one H_2_ molecule adsorbed are shown in Table 2. H1 and H2 represent the two hydrogen atoms of the adsorbed hydrogen molecule, and C2, C3 and C4 are the three C atoms that charge transfer most in the PG layer (as shown in Figure 1). After the first H_2_ adsorption, the charge of each atom of the H_2_ molecule is −0.13 e, while the Sc atom carries the positive charge of 1.56 e. According to Figure 5, Sc 3d orbital and the H_2_ molecule of bonding and antibonding state are hybridizations. Therefore, H_2_ molecule transfers charge to the Sc atom, and then Sc atom back donates charge to the antibonding orbital of the H_2_ molecule. As a result, the H_2_ molecule carries a more negative charge. There is Coulomb attraction between the negatively charged H_2_ molecule and positively charged Sc atom. This is consistent with the case of Ca-decorated graphene for hydrogen storage [42]. The Coulomb interaction enhances the adsorption of the H_2_ molecule. Although the bond length of the H_2_ molecule is stretched, the H_2_ molecule remains its molecular bond. After one H_2_ molecule adsorption, the negative charge of C2 and C4 atoms increase. However, the negative charge of C3 nearest the H_2_ molecule is reduced by about 0.15 e, which indicates that these C atoms also play a role in H_2_ molecular adsorption.

**Figure 6:**
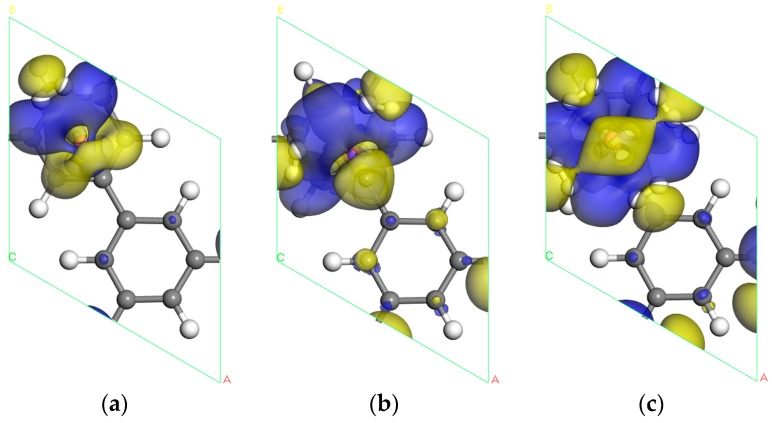
Electronic charge density difference for Sc-decorated PG system in the presence of (**a**) one H_2_ molecule; (**b**) two H_2_ molecules; (**c**) four H_2_ molecules.

The charge transfer of the Sc-PG system can be intuitively observed through charge density difference. Therefore, the charge density difference ∆ρ is defined as:

(4) $$∆\rho =\rho_{iH_{2}+\mathrm{Sc}+\mathrm{PG}}-\rho_{iH_{2}}-\rho_{\mathrm{Sc}+\mathrm{PG}}$$

where $\rho_{iH_{2}+\mathrm{Sc}+\mathrm{PG}}$ is the electron charge density of the total system, $\rho_{iH_{2}}$ is the charge density of *i* adsorbed H_2_ molecules, and $\rho_{\mathrm{Sc}+\mathrm{PG}}$ is the charge density of the Sc decorated PG system. The charge density difference for the system Sc-PG with H_2_ molecules adsorbed is shown in Figure 6. The blue and yellow isosurface represents space charge accumulation and depletion, and the value for the isosurface is 0.007 e/${\text{Å}}^{3}$. It is easy to see that the charge transfer is mainly distributed in regions between H_2_ molecules and the Sc atom, indicating a strong interaction between them. The charge transfer of C atoms in PG is also observed, which indicates that these C atoms also play a role in H_2_ molecular adsorption. It demonstrates the H_2_ molecules are polarized such that charge depletion and accumulation at both ends of H_2_ molecules can be observed in Figure 6b. Combining the Mulliken charge population, each H atom of H_2_ molecules carries a negative charge of −0.16 e and −0.14 e, and it has a Coulomb attraction with a positively charged Sc atom after polarization. Consequently, the adsorption of hydrogen molecules in the Sc-decorated PG system is due to the orbital hybridization among H, Sc, C atoms, and Coulomb attraction between negatively charged H_2_ molecules and positively charged Sc atoms.

### 3.2. Two Sc Atoms Decorated PG 

#### 3.2.1. The Adsorption Structure of Two Sc Atoms Decorated PG

The cohesive energy of Sc atoms is 3.90 eV [43], which is much bigger than the binding energy of Sc atoms on PG. In order to test the possibility of Sc aggregation, we add the second Sc atom in the system on the same side. Figure 2b presents the most stable optimized geometry for two Sc atoms adsorbed on PG. We find that the second Sc atom is more likely to adsorb on the center of another C hexagon rather than aggregate with the first Sc atom, and this is mainly because the Coulomb repulsion between two Sc atoms (both Sc atoms carry 0.87 eV positive charge) and the strong interaction between Sc atoms and PG. The average binding energy of two Sc atoms on PG is −3.195 eV, which implies that the interaction between the second Sc atom and PG is stronger than the first. In addition, the large distance of 4.322 Å between two Sc atoms may be another reason for resistance clusters.

To examine further the space for hydrogen storage, this paper also studies two Sc atoms decorated double-sided PG. Each transition metal atom is an active adsorption point, and transition metal atoms modified double-sided PG can increase the hydrogen storage area. Based on the Figure 2a system, there are four adsorption positions for the second Sc atom on the opposite side of PG. The stable structures after relaxation are shown in Figure 2c,d and the average adsorption energies of the Sc atom are −2.813 eV and −2.537 eV. The hydrogen adsorption energy and storage properties of these two models are investigated in our work since the binding energy of Sc atoms has little difference.

#### 3.2.2. The Hydrogen Storage Capacity of Two Sc Atoms Modified PG System

**Figure 7:**
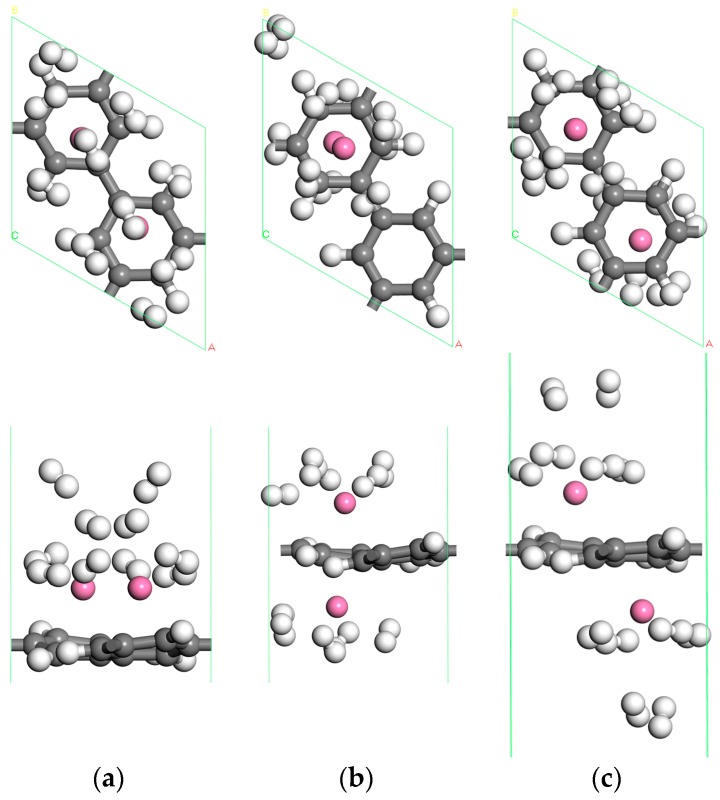
The optimized geometries for H_2_ molecules adsorbed on Sc-decorated PG. (**a**) two Sc atoms decorated single-sided PG; (**b**) two Sc atoms decorated double-sided PG at the same hole; (**c**) two Sc atoms decorated double-sided PG at the adjacent hole.

As shown in Figure 7a, about ten H_2_ molecules can be adsorbed in the two Sc single-sided modified PG system without aggregation. The average adsorption energy is about −0.268 eV/H_2_, and the hydrogen storage capacity is 7.69 wt %. The cohesive energy of the Sc atom is higher than its binding energy on PG, and after H_2_ adsorption the two Sc atoms are slightly closer to each other. This means that Sc aggregation may occur on the porous graphene, but from the optimized structure we not find Sc atom bonding with each other although Sc atoms can move freely. The average binding energy of two Sc atoms on PG is −3.195 eV, which means that the adsorption structure of the two Sc atoms on the PG is more stable than that of a single Sc on the PG. This indicates adsorption structures are stable. After testing, we found that low concentrations of Sc atoms do not aggregate on PG. This phenomenon may be due to the Coulomb repulsion between two Sc atoms and the strong interaction between Sc atoms and PG, as well as the pore structure of the porous graphene. Given that the hydrogen storage space of PG on a single side is limited, to further improve the hydrogen storage capacity, two Sc atoms modified double-sided PG are also considered, as shown in Figure 2c,d. After hydrogen storage, the optimized structures are shown in Figure 7b,c. And the distance between the Sc atoms adsorbed on both sides of PG is large enough to prevent the aggregation. The Figure 7b system can adsorb ten H_2_ molecules, the average adsorption energy of H_2_ molecules is −0.292 eV, and the hydrogen storage capacity is 7.69 wt %. The system of Figure 7c contains twelve H_2_ molecules adsorbed on Sc-decorated both sides of PG. The average adsorption energy of hydrogen in this system is −0.296 eV/H_2_ higher than that of −0.23 eV/H_2_ in the Y-PG system, and the hydrogen storage capacity is up to 9.09 wt %, higher than that of 7.87 wt % in the Y-PG system [35].

The ideal adsorption energy between H_2_ molecules and materials should remain in the range of 0.2~0.7 eV/H_2_, which is between the physical and chemical adsorption state. It will be beneficial to hydrogen storage and release under the environmental temperature and pressure. In our work, the average adsorption energies of H_2_ molecules adsorbed on graphene are from −0.060 to −0.075 eV/H_2_. The adsorption energy is in the range of −0.061~−0.104 eV/H_2_ for H_2_ molecules adsorbed on PG. The adsorption energy of H_2_ molecules at the 4 sites in Figure 1 (in the same plane with PG) is positive and is the most unstable adsorption site. Therefore, the adsorption energy of H_2_ molecules adsorb on intrinsic graphene and PG is very small, which is not conducive to hydrogen storage.

The three systems of two Sc atoms modified PG for hydrogen storage are shown in Figure 7a–c. Their average adsorption energies are −0.268 eV/H_2_, −0.292 eV/H_2_, −0.296 eV/H_2_ respectively, which is small in the ideal range. The systems of Figure 7a,b have the same hydrogen storage capacity, but the average adsorption energy of Figure 7b is bigger than that of Figure 7a. The hydrogen storage capacity of Figure 7c is the largest of the three systems, and the average adsorption energy of H_2_ molecules is the largest. As a result, the system of Figure 7c is the best structure for reversible hydrogen storage.

In addition, in comparing these systems (Figure 4e and Figure 7a,c) we find an interesting phenomenon: The H_2_ molecules in Figure 7a are distributed in three layers, so the average adsorption energy is small (−0.268 eV/H_2_). In Figure 4e and Figure 7c, there are two layers of H_2_ molecules, and the average adsorption energies of −0.429, −0.296 eV/H_2_ are larger. To sum up, two Sc atoms located above the adjacent C ring in double-sided PG is the most suitable structure for hydrogen storage. Therefore, Sc-decorated PG is a promising material for hydrogen storage.

## 4. Conclusions

In summary, based on the density functional theory, we studied the hydrogen storage properties of the Sc-PG system. For a single Sc atom, the most stable adsorption position on PG is the carbon hexagon center with an adsorption energy of −2.143 eV. Four H_2_ molecules can be adsorbed around an Sc atom with an average adsorption energy of −0.429 eV/H_2_. Analyzing the electronic structure of the system, the adsorption of H_2_ molecules in the PG system is mainly attributed to orbital hybridization among H, Sc, C atoms. The largest hydrogen storage capacity structure is two Sc atoms located above the adjacent C ring in a double side of PG. The system can absorb twelve H_2_ molecules with an average adsorption energy of −0.296 eV/H_2_, and the hydrogen storage capacity is 9.09 wt %. Therefore, Sc-decorated porous graphene may become one of the most promising hydrogen storage materials.

## Figures and Tables

**Table 1:** The adsorption energy, average adsorption energy and the nearest distance between Sc and C of porous graphene for H_2_ adsorbed on single Sc decorated PG.

Number of H_2_	$E_{ad}\;$(eV)	${\bar{E}}_{ad}\;$(eV)	$d_{Sc-C\;}$(Å)
1H_2_	−0.401	−0.401	2.348
2H_2_	−0.718	−0.559	2.217
3H_2_	−0.422	−0.514	2.291
4H_2_	−0.174	−0.429	2.451
5H_2_	−0.093	−0.361	2.477

**Table 2:** Mulliken population analysis of the Sc-PG system before and after one H_2_ molecule adsorption.

Atom	Before Adsorption (e)				After Adsorption (e)			
	s	p	d	Charge	s	p	d	Charge
H1	1.00	-	-	-	1.13	-	-	–0.13
H2	1.00	-	-	-	1.13	-	-	–0.13
C2	1.19	3.19	-	–0.38	1.19	3.23	-	–0.42
C3	1.20	3.28	-	–0.48	1.18	3.15	-	–0.33
C4	1.19	3.20	-	–0.39	1.19	3.23	-	–0.42
Sc	0.18	5.84	1.65	1.33	0.04	5.63	1.77	1.56
